# Monitoring unfractionated heparin in ECMO and cardiac surgery: the search for a valid reference standard

**DOI:** 10.3389/fmed.2026.1818646

**Published:** 2026-05-14

**Authors:** Christian F. Weber, Kai Zacharowski, Andreas Calatzis, Marie L. Lindner

**Affiliations:** 1 Department of Anesthesiology, Intensive Care Medicine, Emergency Medicine and Pain Therapy, Schoen Clinic Hamburg Eilbek, Hamburg, Germany; 2 Department of Anaesthesiology, Intensive Care Medicine and Pain Therapy, University Hospital, Goethe University Frankfurt, Frankfurt, Germany; 3 Institute for Cardiovascular Prevention (IPEK), Ludwig-Maximilians-Universität München, Munich, Germany; 4 Department of Anesthesiology, Intensive Care and Emergency Medicine, Asklepios Clinics Hamburg, AK Wandsbek, Hamburg, Germany

**Keywords:** activated clotting time, anti-Xa activity assessment, activated partial thromboplastin time, cardio-pulmonary bypass, dextran sulfate, extracorporeal membrane oxygenation, unfractionated heparin, viscoelastometry

## Abstract

Unfractionated heparin remains the standard anticoagulant during cardiopulmonary bypass (CPB) and extracorporeal membrane oxygenation (ECMO). Despite decades of clinical use, however, no universally validated reference method exists for measuring biologically active unfractionated heparin in these settings. Conventional assays such as the activated clotting time and the activated partial thromboplastin time lack specificity and standardisation. Consequently, anti–factor Xa (anti-Xa) assays are increasingly recommended as alternative methods (or even reference methods). Nevertheless, their validity as a true reference standard in CPB and ECMO has never been formally established. This situation creates a methodological paradox: an assay with context-dependent limitations is used to calibrate and validate other imperfect tests. Anti-Xa assays differ substantially in design, particularly regarding the inclusion of dextran sulphate. Dextran sulphate was introduced to prevent *ex vivo* neutralisation of heparin by platelet factor 4. However, platelet factor 4 release also occurs *in vivo* during extracorporeal circulation. Depending on assay configuration and clinical context, anti-Xa measurements may therefore overestimate or underestimate biologically active heparin—especially at low concentrations or following protamine reversal. As a result, anti-Xa assays cannot be assumed to be interchangeable in CPB and ECMO. We argue that heparin monitoring in extracorporeal circulation requires conceptual re-evaluation. Distinct clinical scenarios must be differentiated, and rigorous validation studies are essential before any assay can be regarded as a true reference standard.

## Introduction

Unfractionated heparin (UFH) is a heterogeneous, sulphated glycosaminoglycan anticoagulant that exerts its effect primarily by potentiating antithrombin, thereby accelerating inhibition of thrombin and factor Xa (1). In cardiac surgery with cardiopulmonary bypass (CPB), UFH is the standard systemic anticoagulant because blood contact with the extracorporeal circuit activates coagulation, and UFH provides rapid, titratable anticoagulation that can be reversed with protamine at the end of bypass (2). For extracorporeal membrane oxygenation (ECMO), UFH is likewise widely used to mitigate circuit- and patient-related thrombotic complications during prolonged extracorporeal support (3).

UFH administration usually includes monitoring and dose adaptation because the required doses in CPB and ECMO show marked interpatient variability, and both under- and over-anticoagulation can lead to clinical complications (2). During CPB, monitoring is commonly performed using the activated clotting time (ACT) in a near-patient setting, sometimes supplemented by heparin concentration–based or anti–factor Xa (anti-Xa)–based approaches (2). In ECMO, either the activated partial thromboplastin time (aPTT), the ACT, or anti-Xa analysis is performed (4).

The limitations of the aPTT and ACT as heparin-monitoring assays are well known and generally accepted. Both assays show poor correlation with actual UFH concentrations as determined by anti-Xa analysis (5–9). This is not surprising given the multitude of factors involved in these assays (contact phase; clotting factors XII, XI, IX, VIII, X, V, and II); therefore, the assay reaction is relatively unspecific for the drug to be tested (heparin). In addition, standardisation of these assays is poor: different aPTT reagents have different sensitivities to UFH, and whole-blood point-of-care aPTT assays do not agree well with laboratory aPTT tests (10–12). Different ACT assays are likewise not interchangeable (6, 13–15). Thus, both assays are analytically weak tools for monitoring UFH and poorly standardised. On the positive side, they are widely available, rapid, and inexpensive.

Viscoelastic testing (VET) is another whole-blood–based coagulation test method that has been proposed as a tool to monitor unfractionated heparin (16–18). The VET assays used for this purpose (CK on TEG, in-tem on ROTEM, and IN-test on ClotPro) are—similar to aPTT and ACT—triggered via the contact phase and thus involve factors XII, XI, IX, VIII, X, V, and II, which renders them relatively unspecific for heparin. In several publications (16, 17), results are expressed as ratios of clotting times before and after inactivation of heparin by heparinase, which may improve assay performance. However, overall clinical experience with these VET-based approaches in ECMO and cardiac surgery remains limited, and current review recommendations are cautious regarding their use in these settings (19, 20). As noted, VET assays are activated via the intrinsic pathway and therefore remain susceptible to the same confounding factors that affect aPTT and ACT, including contact activation, factor deficiencies, haemodilution, and inflammatory alterations. Consequently, their ability to reliably quantify residual heparin effects—especially in the setting of protamine reversal—may be constrained.

Considering these limitations, anti-Xa testing—although more time-consuming and restricted to laboratory-based analysis—has been recommended as an alternative method for heparin monitoring in ECMO and, in selected cases, during cardiac surgery with CPB (2, 21). Given that UFH represents a heterogeneous mixture of sulphated glycosaminoglycan chains with variable lengths and molecular weights (1), direct measurement of the drug (e.g., by mass spectrometry) is not feasible as a reference method.

The 2022 ELSO Adult and Paediatric Anticoagulation Guidelines acknowledge several limitations of anti-Xa assays, including limited standardisation among commercially available tests, interference by dextran sulphate, and potential analytical sources of error. Nevertheless, they provide example target ranges based on anti-Xa activity (21). By contrast, the 2023 ISTH/SSC guideline on anticoagulation in adult patients supported by ECMO does not explicitly address these methodological limitations (22). Similarly, the STS/SCA/AmSECT Clinical Practice Guidelines on anticoagulation during CPB recommend anti-Xa testing as a validating assay for point-of-care tests reflecting anti-Xa activity, yet do not specify how anti-Xa testing itself was validated for this purpose in the CPB setting (2).

Despite these recommendations, a fundamental question remains insufficiently addressed: can anti-Xa assays be considered a validated reference standard for UFH monitoring in CPB and ECMO? If an assay with context-dependent methodological limitations is used to calibrate other imperfect tests, a conceptual circularity arises. This Perspective examines whether current monitoring strategies rest on sufficiently validated premises and outlines a framework for re-evaluating UFH monitoring in extracorporeal circulation.

## Discussion

### Are recommendations to use anti-Xa testing in cardiac surgery and ECMO justified?

A fundamental principle of epistemology is that conclusions cannot be more robust than the premises on which they are based. Assigning reference status to an assay that has not been adequately validated for a specific clinical context is problematic, particularly when subsequent recommendations and target ranges are derived from it.

Anti-Xa assays were not originally developed for monitoring UFH during cardiac surgery or ECMO, and several uncertainties remain regarding their suitability in these settings (23). Although anti-Xa assays are generally considered better standardised than aPTT or ACT (24), inter-assay variability between commercially available tests has been documented (25, 26). A key determinant of assay behaviour is the use of dextran sulphate (24), which is included in some reagents (HemosIL® Liquid Anti-Xa by Werfen, INNOVANCE® Heparin by Siemens, and BIOPHEN™ Heparin LRT by Hyphen) but not in others (STA® Liquid Anti-Xa by Stago, Technochrom® Anti-Xa by Technoclone).

The rationale for introducing dextran sulphate into certain anti-Xa assays was to prevent *ex vivo* inhibition of heparin during sample handling (24, 27). During blood transport, storage and centrifugation to obtain platelet-poor plasma, platelet activation may occur, resulting in the release of platelet factor 4 (PF4). PF4 is a platelet-derived protein capable of binding to and neutralising the biological activity of heparin (28). Dextran sulphate was therefore added to some anti-Xa reagents to displace heparin from PF4 and to avoid falsely low anti-Xa results caused by *ex vivo* heparin neutralisation (24, 27).

However, the use of dextran sulphate in anti-Xa testing has recently been questioned (23, 29), particularly because it may lead to overestimation of heparin concentrations, e.g., after protamine administration (29, 30). Importantly, PF4 release is not limited to *ex vivo* sample handling. Platelet activation with subsequent PF4 release occurs *in vivo* during CPB and ECMO support (31, 32). In these settings, dextran sulphate–containing anti-Xa assays may liberate heparin that has already been neutralised in vivo by PF4, or subsequently by protamine, and render it measurable *in vitro*. This mechanism may result in falsely elevated anti-Xa levels that do not necessarily reflect biologically active circulating heparin.

Conversely, anti-Xa assays that do not contain dextran sulphate may be susceptible to *ex vivo* PF4-mediated heparin neutralisation during sample transport and processing. This may lead to falsely low anti-Xa results, particularly when pneumatic tube systems, centrifugation delays, or prolonged pre-analytical intervals are involved. Thus, depending on assay composition, anti-Xa testing in cardiac surgery and ECMO may be prone either to overestimation or underestimation of clinically relevant heparin activity. Switching to currently available point-of-care assays such as ACT, whole-blood aPTT, or VET does not appear to resolve these fundamental methodological problems, for the reasons outlined above.

A mechanistic comparison of the assays applied for the monitoring of heparin is shown in Table 1.

**Table 1:** Mechanistic comparison of assays used for the monitoring of UFH.

Monitoring method	POC	Specificity for heparin	Sensitivity for heparin	Interference with protamine	Inactivation of heparin during sample transport and preanalytics
Anti-Xa with DS	No	High^2^	High	Potentially yes^6^	Unlikely (DS)
Anti-Xa without DS				No	Potentially yes^7^
aPTT	Mostly no^1^	Low^3^	Moderate	Yes (prolongation when protamine is present in excess)	
ACT	Yes		Moderate/low ^4^		No (POC)
VET in-tem/IN-test			Moderate		
VET CK (TEG)			High^5^		

^1^POC aPTT tests exist, most aPTT tests are however laboratory-based.

^2^Detection of UFH action via the inhibition of FXa or FIIa (thrombin).

^3^Detection of UFH action in an assay reaction mediated via factors XII, XI, IX, VIII, X, V, II.

^4^The ACT is usually optimized for a wide measuring range for the detection of the high heparin doses applied in cardiac surgery.

^5^The CK assay uses a much weaker intrinsic activation as compared to aPTT or in-tem/IN-test.

^6^Several publications claim a release of protamine-bound heparin by dextrane sulphate.

^7^Platelet activation during sample transport, centrifugation and waiting periods could lead to release of PF4 and subsequent *ex vivo* heparin inactivation.

### What are the consequences?

If anti-Xa assays are to be used as reference methods in cardiac surgery and ECMO, their methodological limitations must be explicitly acknowledged.

First, no universally validated reference assay currently exists for the assessment of UFH activity during and after CPB or ECMO support. Second, pending appropriately designed comparative studies using samples derived specifically from CPB and / or ECMO patients, different anti-Xa assays cannot be considered interchangeable in these settings. Reported target ranges and inter-study comparisons must therefore always be interpreted considering the specific reagent and assay configuration applied. Third, the impact of dextran sulphate on anti-Xa measurements in cardiac surgery and ECMO requires systematic investigation, particularly in the context of protamine reversal and low residual heparin concentrations.

In theory, a potential reference strategy could involve the use of an anti-Xa assay without dextran sulphate in combination with blood collection in citrate–theophylline–adenosine–dipyridamole (CTAD) tubes, which may reduce *ex vivo* platelet activation and PF4 release (33). However, such an approach would itself require formal validation within the specific clinical contexts of CPB and ECMO before it could be considered a reliable standard.

### Distinguishing clinical scenarios

Importantly, monitoring strategies for UFH cannot be discussed as a single homogeneous problem. At least four distinct clinical scenarios should be differentiated:

- Standard-dose UFH outside extracorporeal circulation (e.g., treatment of deep vein thrombosis):
  In this setting, significant *in vivo* PF4 release is unlikely and protamine is not administered. Dextran sulphate may therefore be appropriate because PF4-bound heparin would predominantly reflect *ex vivo* phenomena rather than *in vivo* (“true”) neutralisation.
- High-dose UFH during CPB:
  Here, very high heparin concentrations are present and in vivo PF4 release occurs. Given the magnitude of circulating heparin levels, the quantitative impact of dextran sulphate–mediated heparin release is likely to be negligible in relation to the total measurable activity.
- Low- to moderate-dose UFH during ECMO:
  In this scenario, *in vivo* PF4 release is expected, while heparin concentrations are substantially lower than during CPB. Under these conditions, dextran sulphate–mediated displacement of PF4-bound heparin may meaningfully influence (and potentially distort) measured anti-Xa levels.
- Detection of residual UFH after protamine administration:
  When low residual heparin concentrations are to be assessed, liberation of protamine-bound or PF4-bound heparin by dextran sulphate may lead to clinically misleading results. Recent reports (29, 30) suggest that this risk warrants careful evaluation.

The suitability of the different monitoring methods for these clinical scenarios is summarized in Table 2.

**Table 2:** Suitability of heparin monitoring methods for different clinical scenarios.

Monitoring method	i.v. Heparin without bypass (e.g., ICU)	Cardiac surgery (before protamine)	Cardiac surgery (after protamine)	ECMO
Anti-Xa with DS	++ DS reducing preanalytical errors due to heparin inactivated *ex vivo*	o POC ACT is an established procedure for this use, anti-Xa is mainly proposed as a tool for validating the ACT method applied, due to time delay and cost	o Interpretation may be limited due to potential interference of dextran sulphate with protamine–heparin and PF4–heparin complexes formed *in vivo*	o interpretation may be limited due to potential interference of dextran sulphate with protamine–heparin and PF4–heparin complexes formed in vivo
Anti-Xa without DS	+ Potential inactivation of heparin during preanalytics		+ potential inactivation of heparin during preanalytics	
ACT	o Weak dose–response for heparin	+ Established procedure	o Due to low expected residual heparin doses the assay specificity/sensitivity for heparin might be suboptimal	o the assay specificity/sensitivity for heparin might be suboptimal in ECMO
aPTT		− No clotting due to high heparin concentrations		
VET in-tem/IN-test				
VET CK (TEG)				

DS, dextran sulphate; POC, point of care; PF4, platelet factor 4; ACT, activated clotting time; VET, viscoelastic testing; ECMO, extracorporeal membrane oxygenation.

++ = highly suitable; + = suitable with limitations; o = limited suitability; − = not suitable.

### A research agenda

For each of these clinical indications, three fundamental steps are required:

- Definition of an appropriate reference method for UFH measurement
- Establishment of clinically meaningful acceptance criteria
- Validation of candidate monitoring tools (including point-of-care assays) against these predefined standards

Once such suited heparin monitoring methods have been characterized, their results would have to be investigated in relation to the clinical course including thrombotic or hemorrhagic events, with particular emphasis on their performance during prolonged CPB or ECMO, conditions which are known to be associated with a progressing activation of coagulation.

This effort will require coordinated, multicenter clinical studies and close collaboration across disciplines. Six decades after the introduction of the ACT (34), reliable monitoring of UFH in extracorporeal circulation remains an unresolved challenge.

## Data Availability

The original contributions presented in the study are included in the article/supplementary material, further inquiries can be directed to the corresponding author.
